# Biophysics - whence, whither, wherefore - or Hold that hyphen

**DOI:** 10.1186/1741-7007-9-12

**Published:** 2011-03-02

**Authors:** Walter B Gratzer

**Affiliations:** 1 Randall Division of Cell and Molecular Biophysics, New Hunt's House, King's College London, Guy's Campus, London, SE1 1UL, UK

## 

In 1961 the American Chemical Society announced the launch of a new journal: it was to be called *Bio-chemistry*. A hyphen of course has its place - think for instance of the headline in a local newspaper: 'Police Help Dog Bite Victim' - but *Bio-chemistry *caused outrage and derision (I was there). Why return to the usages of the 19th century, biochemists (and others) wanted to know. The American Chemical Society drew back, and it was as *Biochemistry *that the journal hit the news-stands the following year. All the same, one could see what was in the editors' minds. They were not proposing to compete for papers with the estimable *Journal of Biological Chemistry*, for their new organ was meant as an outlet for research in a branch of chemistry. Biophysics too had by then been liberated from its hyphen, with the implication that it was to be regarded as a mature discipline. And yet it was and remains a hybrid. Francis Crick long ago instructed us that we were living in an interdisciplinary age. There was almost no branch of science that could not be conjoined with something seemingly quite alien: astrobotany was his favorite example of a field of study yet to emerge.

It was well into the 19^th ^century that men of science - the term 'scientist' was still grossly offensive to a classically educated generation - came to identify themselves with specialisms. The likes of Newton, Hooke, and later Davy and Faraday made no distinction between physics and biology: Hooke did public physiological demonstrations, Newton developed a theory of nerve action and Faraday measured the magnetic properties of a beefsteak. Hooke, Boyle, the Reverend Stephen Hales and many others sought to interpret physiological processes in terms of physical laws and with the help of physical apparatus. The discovery of static electricity caused an ebullition without precedent of public interest, and demonstrations of its effects attracted crowds. Benjamin Franklin 'fetched lightning out of the sky' down a metal wire attached to a kite. His rival, the Abbé Nollet, lined up more than a hundred Carthusian monks, told them to hold hands, connected a wire to the two at the ends, and applied a discharge from a Leyden jar. The observers were thrilled to see them leap in unison and utter a synchronized cry of alarm. Not long after, biophysics acquired a martyr, when Professor GW Richmann of the University of St Petersburg repeated Franklin's experiment, exclaiming that in these hard times even a physicist could display courage, and was struck dead.

The theory of 'animal electricity' - of an 'electric fluid', which flowed along the nerves - received its impetus from Galvani and his twitching frogs' legs. It inspired Mary Shelley to write about Frankenstein, and provoked attempts to revive the dead by electric means. It took a physicist, Alessandro Volta to show that there was no electric fluid, and that Galvani had merely set up a bimetallic cell. This did not deter Galvani's nephew, Giovanni Aldini, who crouched at the foot of the guillotine, poked electrodes into the brains of the severed heads, and found that their eyes opened and their lips grimaced. The far more scientific Alexander von Humboldt experimented on himself, even pushing an electrode into the cavity left by an extracted tooth. (The pain was great and the consequences minimal.) These men could be seen as the progenitors of today's biophysicists.

In the mid-19^th ^century the great French physiologists Magendie and Bernard put an end to vitalism, and insisted that the laws of chemistry and physics must account for all biological phenomena. The term biophysics probably made its first appearance in Germany in the mid-19^th ^century in the writings of a group of remarkable polymaths, most famously Helmholtz and Du Bois-Reymond, for whom it signified a rational analytical approach to physiology. Biophysics, though, became a profession much later. The first textbook in English with biophysics in its title that I have been able to find appeared in 1921. The author of *An Introduction to Biophysics *was David Burns, Lecturer in Physiological Chemistry at the University of Glasgow. The Introduction is by DN Paton, Regius Professor of Physiology, who concedes that chemistry and physics have impacted on his subject, but goes on to grumble about the predilection of his older brethren for making uninterpretable observations and giving them 'high-sounding Greek names'. 'The so-called chemical physiologists', he petulantly asserts, 'are perhaps the worst offenders. For, having isolated, or thought they had isolated, some constituent of the body of quite unknown chemical constitution, they promptly gave it a name with no connection with its chemical nature...... In the present age of "hormones" and "vitamines" one wonders how far this tendency has been eradicated'. Such were the concerns in the British universities. (It is true however that vitamin, or vitamine, standing for 'vital amine', is a misnomer.) The learned professor does confess that intrusion of 'the more exact science of physics, based as it is largely upon mathematics' has helped to discourage 'vague theorising'. The contents of the book are of course directed at physiologists and medical students. The chapters are Energetics, Cellular mechanisms, Cell communities, Transport (meaning blood, digestion and so on), and The Animal as a whole. There is little mention of molecules, and the substance of all chapters but the first has receded into the mists of history.

Biophysics was seen, then, as a prop for the serious business of physiology, and it later also became conflated with medical physics, which essentially meant radiology. Hospital physicists would have ranked low in the medical hierarchy and in the esteem of the physics fraternity. The Olympians, such as Bohr (with his *obiter dicta *on the implication of the Complementarity Principle for biology) and Schrödinger, ruminated languidly on the nature of life, but biologists who, as Peter Medawar put it, 'operate at the frontier between bewilderment and understanding', were not generally regarded in such quarters as altogether scientifically house-trained. The Victorian physicist PG Tait spoke of 'minds debauched by the so-called science of biology', while for Rutherford there were only physics and stamp-collecting. But then one of their own, no less than Erwin Schrödinger, came out with a slim volume with the modest title, *What is Life? *It appeared in 1944, a few months before the end of the Second World War, and it received close attention from physicists and physical chemists, many of them wearied by years in war work, and in want of a fresh outlet for their talents. It is remarkable indeed how many of the founders of the new biology were animated by Schrödinger's little book. For its message was that biology really was physics, despite the apparent conflict between life and thermodynamic imperatives, and especially that the vehicle of heredity, so far from being a kind of intangible essence, would turn out to be an 'aperiodic crystal'. The concept was never properly defined, but it carried the alluring implication that it might be open to study by established physical methods, most obviously X-ray diffraction.

It was only much later that some of those who had been captivated by Schrödinger's dissertation began to wonder why they had so uncritically swallowed it all. Max Perutz reflected in 1987 on the author's sleight of hand: 'A close study of the book and of the related literature has shown me that what was true of the book was not original, and most of what was original was known not to be true even when it was written'. More, 'the apparent contradiction between life and the statistical laws of physics can be resolved by a science largely ignored by Schrödinger. That science is chemistry'. Perutz's strictures, it should be said, did not go unchallenged, and drew, in particular, a lucid response from an eminent geneticist and quondam associate of Schrödinger's, Neville Symonds. At all events there is no doubting the book's effect in making biophysics attractive to many and at least halfway respectable. It is curious though that its rise was prefigured in a novel, published in 1934: *The Search *by CP Snow has for its hero a visionary young physicist who procures funding to set up in London, at a location plainly King's College, a department of biophysics.

In England it was JD Bernal whose eloquence and authority had the greatest influence in establishing the new subject in the postwar decade. Soon biophysics departments and units were springing up like mushrooms. In 1947 WT Astbury hailed 'the dawn of a new era' and proclaimed that 'there is an urgency for more intensive application of physics and chemistry, and especially of structural analysis, that is still not sufficiently appreciated'. And yet the question remained: what exactly *is *biophysics. The first volume of *Progress in Biophysics and Biophysical Chemistry *appeared in 1950, and the editors' preface betrays a certain unease. They had had, they confessed, 'some difficulty in deciding what is the proper field to be covered by reviews of recent progress in biophysics. Excluding biochemistry on the one hand and physiology on the other, there lies between a vast and rather amorphous field of study of which the frontiers and lines of demarcation are anything but well defined'. There follow more comparisons of what might lie on the one hand and what on the other, reminiscent of the politician who wished for a one-armed economist, but little in the way of conclusions. The contents are in fact remarkably similar to those of other series starting up around the same time, all in general strong on radiation effects and the use of isotopes.

One of the editors, a professor of physics, had established a biophysics unit, and sought at one point to recruit a biologist who would generate materials for the physicists to study. The biologist in question related to me a telling vignette of the kind of thinking, not uncommon at the time. As the interview progressed, the professor leaned back in his swiveling chair, picked his nose, and rubbed the product between first finger and thumb. 'You see', he said, 'you can pull fibers from this, do X-ray diffraction, get the structure'. Here at least was a biophysicist willing to prepare his own material, for it was a common criticism that physicists entering biology were too often reluctant to get their hands wet, or even bloody. They preferred instead to work on material bought or begged from other laboratories, a trait not unknown among their successors today. But as time passed, the boundaries between disciplines became ever more blurred. Much of physiology, most clearly in the monumental studies of nerves and muscle, became pervaded by the methods of physics and physical chemistry. Then in the early 1960s a chic new calling arose: molecular biology, derided by Chargaff as biochemistry practiced without a license, was indeed created in the main by physicists and physical chemists. Much of it centered, as Astbury had foreseen, on molecular structure.

There were, to be sure, many curious cul-de-sacs along the route. Crick disapproved of mathematicians who swam into his orbit: he thought them intellectually lazy because unwilling to immerse themselves in the science they claimed to illuminate. The same could be said of many of the physicists who had invaded biology and were discovering conduction bands in proteins baked at 250°C, working out the biological implications of proton tunneling in DNA or calculating the torsional modes of its backbone. They bombarded animals and molecules with ionizing radiation, and they searched for formation of charge transfer complexes by carcinogens. A mathematician, Dorothy Wrinch, put forward an improbable scheme of geometrical hierarchies in living organisms, and especially the misbegotten 'cyclol' theory of protein structure. This was grounded in a rejection of the amide bond we know in favor of a ring configuration of its enolic state. The theory had for a time adherents in high places, notably the great physical chemist Irving Langmuir. It was promoted in a book about protein structure and dynamics, published after X-ray crystallography had actually exposed peptide bonds to view in real polypeptides.

Accompanying the rise in quantitative rigor and technical sophistication of biological research in our time has been the fading of 'physics envy', once described as the curse of biology. Instead, the physicists, along with chemists and engineers, are surging into biology. This has rejuvenated both the biological and the physical sciences, even if the leading physics journals now publish a profusion of poorly refereed papers whose authors have not followed the excellent precept not to think what one wants to think until one knows what one ought to know. Nevertheless, biophysics (however defined) has made dizzying advances. As to what lies ahead, best perhaps to heed Niels Bohr's admonition that prediction is very difficult, especially of the future.

